# Probiotic and Technological Potential of Native Lactic Acid Bacteria Strains from High Andean Forest Bee Bread: In Vitro Study

**DOI:** 10.1007/s11130-025-01392-x

**Published:** 2025-10-03

**Authors:** Camila Bernal-Castro, Ángel Camargo-Herrera, Carolina Gutiérrez-Cortés, Consuelo Díaz-Moreno

**Affiliations:** 1 Instituto de Biotecnología (IBUN), Universidad Nacional de Colombia, Bogotá, Colombia; 2 Facultad de Ciencias Agrarias, Universidad Nacional de Colombia, Bogotá, Colombia; 3 Escuela de Ciencias Agrícolas, Universidad Nacional Abierta y A Distancia (UNAD), Pecuarias y del Medio Ambiente (ECAPMA), Bogotá, Colombia; 4 Instituto de Ciencia y Tecnología de Alimentos (ICTA), Universidad Nacional de Colombia, Bogotá, Colombia

**Keywords:** Beekeeping products, Microbiota, Lactic fermentation, Functional properties, Prebiotics

## Abstract

Bioprospecting of lactic acid bacteria with probiotic potential from apicultural products is an important key for the research in functional foods. The in vitro evaluation of the probiotic and technological potential of commercial HOWARU strains and native strains isolated from bee bread was conducted in this study. The strains were molecularly identified (16S rRNA sequencing), revealing differences between molecular characterization and the microorganisms described in the technical datasheet. Most native strains belong to the genus Pediococcus. The ability to resist simulated gastrointestinal conditions (acidic pH and bile salts), as well as tolerance to extreme conditions (high temperature and osmotic pressure), was determined. VEGE 092 culture showed survival levels above 80%, and Pediococcus pentosaceus exceeded 95%. Finally, growth on alternative substrates (by-product of supercritical fluid extraction of bee pollen, car-rot waste flour, and turmeric flour) was evaluate by the quantitative prebiotic index. This study demonstrated that the best symbiotic combination was VEGE 092 and turmeric (prebiotic index = 0.96), and *P. pentosaceus* with the pollen extraction by-product, demonstrating a strain-substrate relationship. This study highlights the potential use of these strains in functional food applications, emphasizing their resilience and ability to thrive in various substrates.

## Introduction

Lactic acid bacteria (LAB) encompasses a group of Gram-positive bacterium characterized by their ability to ferment carbohydrates predominantly into lactic acid as the principal metabolic end product [1]. They are extensively applied in the fermentation of dairy, meat, vegetable, and beverage products, where they significantly influence the sensory attributes and enhance the nutritional profile of the final products [2]. These bacteria are primarily used in the fermentation processes for their metabolic activity by producing a variety of enzymes, including proteases, peptidases, ureases, lipases, amylases, esterases, and phenol oxidases, which are responsible for breaking down polysaccharides, proteins, and lipids and they generate bio-compounds like lactic acid, short-chain fatty acids, carbon dioxide, hydrogen peroxide, lactoperoxidase, diacetyl, and bacteriocins [3].

Probiotics are defined as “live microorganisms that, when administered in adequate amounts, confer health benefits to the host” [4]. These benefits are achieved through mechanisms such as promoting the growth of beneficial microbiota, modulating both mucosal and systemic immune responses, and contributing to the maintenance of microbial and nutritional homeostasis within the gastrointestinal tract (GIT) [5, 6].

Probiotics are predominantly associated with the genera *Lactobacillus* and *Bifidobacterium*; however, other bacterial taxa such as *Bacillus* and *Lactococcus* species, as well as certain yeasts like *Saccharomyces boulardii*, may also exhibit probiotic properties [3]. Recently many researches have increasingly focused on isolating microorganisms from diverse ecological niches to identify novel probiotic candidates among them, *Pediococcus pentosaceus*, a species of lactic acid bacteria, has garnered considerable interest due to its broad spectrum of probiotic properties and its remarkable adaptability and functionality across a range of environmental conditions [7].

The challenge in the biotechnological field is to deliver viable cells to the GIT through effective formulation methods that maintain probiotic stability [8]. A probiotic candidate must withstand stress factors in food processing, such as fermentation, harvesting, lyophilization, variations in temperature, pH, oxidative and osmotic stress during storage Additionally, a probiotic strain should thrive on a wide variety of substrates to generate symbiotics and produce postbiotic substances like organic acids and antimicrobial agents [9].

Bee bread is a natural product obtained from the fermentation of bee pollen mixed with bee saliva and flower nectar within the honeycomb cells of a beehive [10]. It is considered a functional product with high nutritional value (carbohydrates, proteins, and unsaturated fatty acids) and various bioactive molecules (provitamin A carotenoids) [11]. However, a distinguishing compound in bee bread compared to pollen is lactic acid, which is present in higher concentrations than in pollen and contributes to the product’s acidic pH [12] due the abundance of microorganisms in bee bread that affects the chemical composition of bee bread may endow it with probiotic properties [11].

Several studies have investigated the presence of LAB species in pollen and bee bread samples although they are among the most underestimated products derived from bees [11]. There is still limited knowledge about the strains naturally present in High Andean forest bee bread. This traditional product represents a unique ecological reservoir where LAB may have adapted to particular environmental and nutritional conditions. However, their functional properties remain poorly characterized. Based on this gap, our hypothesis is that native LAB strains isolated from High Andean forest bee bread may exhibit distinctive probiotic attributes therefore. The aim of this study was to evaluate the probiotic, technological potential, and growth on alternative substrates under in vitro conditions of native and commercial strains isolated from high Andean forest bee bread.

## Materials and Methods

### Growth Conditions of Commercial Strains

The commercial strains evaluated in this study were: *Bifidobacterium lactis* HN019 (HOWARU^®^ BIFIDO), *Lactobacillus rhamnosus* HN001 (HOWARU^®^ RHAMNOSUS), *Lactobacillus acidophilus* (HOWARU^®^ DOPHILUS), and a mixed culture VEGE 092 (*Lactobacillus acidophilus*,* Lactobacillus paracasei*, and *Pediococcus pentosaceus*). These strains were supplied by IFF Colombia. Reconstitution was carried out in MRS broth (Oxoid, United Kingdom). The cultures were then incubated under anaerobic conditions at 37 °C for 48 h. Subsequently, an aliquot containing the strain was inoculated into a sterile 15 mL Falcon tube with MRS broth and incubated under the previously described conditions. Growth, morphology, and culture purity were verified using Gram staining. A stock of each strain (50 vials) was prepared from the activated cultures, ensuring the same cell line is use for subsequent activities.

### Collection of Bee Bread Samples

The bee bread samples from the stingless bee (*Apis mellifera*) were collected from an apiary in the municipality of Mosquera, at the Herrera Lagoon, during the months of July and August 2021. The honeycomb cell was storage inside a sterile falcon tube before the transport to the Microbiology Laboratory at the Institute of Food Science and Technology (ICTA) from the Universidad Nacional de Colombia (Bogotá). Inside the laminar flow hood, fresh bee bread was collected from the honeycomb cell using a sterile spatula and stored in a sterile container at 4 °C for subsequent analysis.

### Isolation of Lactic Acid Bacteria from Bee Bread Samples

The isolation method suggested by [13] with modifications. One gram of bee bread was mixed with 9 mL of synthetic broth: MRS supplemented with 10% (w/v) bee bread, with the aim of adapting the lactic acid bacteria (LAB) present in the sample [14, 15]. The samples were incubated for 48 h at 37 °C. Subsequently, they were individually cultured on MRS agar (Oxoid, United Kingdom) using the streak plate method. Then, were anaerobically incubate for 48 h at 37 °C in anaerobic jars with an anaerogenic package (Thermo-Fisher, USA). Bacterial colonies from each medium were selected and sub-cultured to obtain pure isolates before being stored at 4 °C. The isolated bacteria were stored after Gram staining and preserved in a glycerol solution at −80 °C.

### Molecular Identification of Lactic Acid Bacteria Strains

The molecular identification of the commercial strains and the isolates from the bee bread samples was performed. Each strain was isolated on MRS agar, and the samples were coded for DNA isolation and purification, as well as the amplification of the 16 S ribosomal gene using PCR (Polymerase Chain Reaction) and subsequent purification of PCR fragments and sequencing by the Sanger method, using the primers 337 F, 518 F, 800R, and 1100R of the 16 S ribosomal gene at CorpoGen Corporation. The amplification of the fragments was verified by electrophoresis and sent to Macrogen Inc^®^ (Korea) for sequencing. The taxonomic analysis of the sequence was performed using the BLAST (Basic Local Alignment Search Tool) tool of the NCBI (National Center for Biotechnology Information), comparing against the reference RNA database “refseq_rna”. Finally, phylogenetic trees were generated using the Tamura-Nei (TN93) genetic distance model, the “Neighbor-Joining” method, and the “Bootstrap” method with one thousand replicates.

### *In Vitro* Evaluation of Probiotic Potential

#### Bile Salt Tolerance

The methodology proposed by [16] with modifications was followed. The bacteria were cultured for 18 h in MRS broth (10 mL), centrifuged at 6000 g, and resuspended in sterile saline solution (0.86%, w/v). Then, 2% (4 mL) of the suspension was transferred to 200 mL of MRS broth containing 0.3% bile salts (SIGMA, USA) and incubated at 37 °C for 3 h. The control treatment was MRS broth without bile salts. After 0, 1, 2, and 3 h of incubation at 37 °C, viable cells were determined by plate counting on MRS agar. The experiments were performed in duplicate for each independent bacterium.

#### Acid Tolerance

The methodology proposed by [16] with modifications was followed. To examine the survival rate of different strains under acidic conditions, 2% (4 mL) of each bacterial suspension prepared in sterile saline solution was transferred to 200 mL of MRS broth adjusted to pH 2.5 and incubated at 37 °C for three hours. The control treatment was MRS broth with unadjusted pH (6.5). After 0, 1, 2, and 3 h of incubation at 37 °C, viable cells were determined by plate counting on MRS agar. The acid tolerance experiments were performed in duplicate for each independent bacterium.

### *In Vitro* Evaluation of Technological Potential

#### High Temperature Tolerance (Heat Shock)

High temperature tolerance was determined using the method described by [17] with modifications. Bacterial strains were activated in 10 mL of MRS broth by incubating for 24 h at 37 °C. They were then centrifuged at 9,000 *g* for 10 min and resuspended in sterile saline. The inoculum was transferred to MRS broth adjusted to an approximate concentration of 8 log CFU/mL. The inoculated MRS broth was incubated in a thermostatic bath (Memmert) at 80 °C for 10 min. Bacterial strain viability was quantifying by plate counting on MRS agar, and the survival percentage was calculated.

#### High Osmotic Pressure Tolerance

The ability of bacteria to tolerate high osmotic pressure was evaluated according to the methodology suggested by [18] with modifications. MRS broth was supplemented with different concentrations of NaCl (2, 4, 6, and 8%). Four milliliters of the medium with standardized cell suspension (2%, v/v) of the strains were separately inoculated and aerobically incubated at 37 °C for 24 h, during which bacterial viability was evaluated at 0 and 24 h by plate counting. The culture medium without NaCl served as the control. The experiments were performed in duplicate for each independent bacterium.

### Growth of Lactic Acid Bacteria Strains on Alternative Substrates

The growth of lactic acid bacteria strains on alternative carbon sources of plant and apicultural origin was evaluated through the prebiotic potential methodology suggested by [19] with modifications. The alternative substrates (turmeric, carrot flour, and bee pollen by-products) were selected based on previous studies from our research group and their recognized bioactive potential, particularly their content of fibers, polyphenols, and other compounds that may support beneficial microbial growth and act as prebiotic sources. The prebiotic index of turmeric (*Curcuma longa*), carrot flour (from vegetable pulp wastes), and the by-product of supercritical fluid extraction of bee pollen were evaluated. Commercial prebiotic, fructooligosaccharides Orafti^®^ P95, with 95% purity (Beneo-Orafti, Belgium), was used as a comparative standard and analytical grade lactose. The growth of probiotics was evaluated after 24 h at 37 °C in a culture medium composed of 10 g/L of lactose (Scharlab, Spain), as control, or one of the four prebiotic potential ingredients under study, 3 g/L of yeast extract (Oxoid, United Kingdom), and 5 g/L of casein peptone (Oxoid, United Kingdom). The number of colony-forming units (CFU) was determined by plate counting on MRS agar (Oxoid, United Kingdom).

This index is the ratio between the growth of the probiotic in the presence of a prebiotic candidate and the growth of a lactic acid strain with probiotic potential in the presence of a control carbon source (lactose). The prebiotic index was calculated according to Eq. 1. When this ratio is greater than 1, it means that the carbohydrate has a positive effect on probiotic growth.

1 $$\mathbf I\boldsymbol\,\mathbf p\mathbf r\mathbf e\mathbf b=\frac{\text{CFU of probiotics in prebiotic carbohydrate}}{\text{CFU of probiotics in control carbohydrate}}$$

### Statistical Analysis

To determine significant differences (*p* < 0.05), one-way ANOVA and generalized linear model tests were performed. The statistical analysis was conducted using Minitab Statistical Software (Version 18, Minitab, Pennsylvania, USA). All assays were conducted under identical experimental conditions for both native isolates and the commercial reference strain, with no differences applied in the testing procedures. This approach ensured direct comparability of the results between strains. Also All assays were performed using three independent biological replicates, each obtained from separately grown cultures under the same experimental conditions. Within each biological replicate, measurements were carried out in triplicate as technical replicates.

## Results and Discussion

### Molecular Identification of Lactic Acid Bacteria

The determined 16 S ribosomal DNA sequences of the eight microorganisms were directly compare with the BLAST database. A high level of similarity in the nucleotide sequences of the 16 S ribosomal DNA (98 to 100% matches) was obtained for both commercial and native isolated strains, as shown in Table 1, which also indicates the length of the compared sequences.

**Table 1:** Molecular identification of bacterial strains

Strain code	Strain origin	Most Closely Related Strain	Sequence Length and Similarity
72 − 1	Isolated from bee bread	*Pediococcus pentosaceus*	1530 (99%)
72 − 2		*Lactiplantibacillus plantarum*	1521 (99%)
22-(1)		*Pediococcus pentosaceus*	1536 (99%)
22-(2)		*Pediococcus pentosaceus*	1555 (99%)
34 − 4	Commercial origin of the HOWARU^®^ line	*Lactobacillus plantarum*	1534 (100%)
34 − 2		*Pediococcus pentosaceus*	1296 (98%)
34 − 3		*Lactobacillus rhamnosus*	1528 (99%)
34 − 1		*Lactobacillus rhamnosus*	1505 (99%)

The taxonomic relationship was established by comparing the sequence in the National Center for Biotechnology Information (NCBI). Similarity to the sequence of the closest type strain is shown as a percentage within parentheses. Commercial bacteria were coded as follows: 34-4 (*L. acidophilus*), 34-2 (VEGE 092), 34-3 (*B.* *lactis*), 34-1 (*L. rhamnosus*)

The strains isolated from the bee bread sample predominantly corresponded to the species *Pediococcus pentosaceus*, with one strain identified as *Lactiplantibacillus plantarum*. The results of this preliminary study are consistent with the literature, which reports that bee bread is a source of lactic acid bacteria such as *Apilactobacillus kunkeei*,* Lactiplantibacillus plantarum*,* Fructobacillus fructosus*,* Levilactobacillus brevis*,* Lactobacillus delbrueckii*,* Lactobacillus musae*,* Lactobacillus crustorum*, and *Lactobacillus delbrueckii* [11]. However, as far is for our knowledge we are one of the first to report the presence of *P. pentosaceus* in bee bread samples from the Andean Colombian forest.

According to FAO and WHO (2017), for lactic acid bacteria (LAB) to be classified as probiotics, the species characterization of the strain must be conducted to validate its functional properties [20]. Different bacterial genera are observed in bee bread, with variation in the relative abundance of dominant taxa in the different layers of the product and types of ecosystems [21]. Regarding the main bacterial genera found in bee bread, lactobacilli appear to be the most abundant, but representatives of the genera *Bifidobacterium*,* Pseudomonas*,* Serratia*,* Bacillus*,* Sphingomonas*,* Fructobacillus*, and *Enterococcus* have also been found [21].

Recently, several strains of *P. pentosaceus* were identified through genetic (16 S rRNA) and phenotypic analyses (carbohydrate fermentation patterns, acidification kinetics, biofilm formation in microplates, and bacteriocin production assay) from pollen grains [22]. However, it is uncommon to find homofermentative lactic acid bacteria (*P. pentosaceus*) in bee bread samples because the main species of lactic acid bacteria in this type of product belong to a special group of LAB defined as frutophilic lactic acid bacteria (FLAB) [23]. The presence of *P. pentosaceus* in Andean forest bee bread is particularly noteworthy, since this matrix is usually dominated by fructophilic LAB. Its ability to persist in this niche may be linked to specific physiological adaptations, such as tolerance to acidic and osmotic stress or efficient metabolism of the available carbohydrates, which could explain its uncommon occurrence in bee bread ecosystems.

For the commercial strains, as can be observed, the microorganisms identified are not the same as those reported by the manufacturer in the probiotic culture technical sheet (Supplementary Materials). Similar results have been reported in isolates from fermented and encapsulated dairy foods [19]. Another study on commercial fermented products found that the labels of beverages and supplements did not match the species names reported on the label, demonstrating that products listing the same species often contain strains with different sequences and phenotypes [24]. This study highlights that the current labelling and technical sheets of probiotics do not adequately convey the strain diversity in these products.

### *In Vitro* Evaluation of Probiotic Potential

**Fig. 1:**
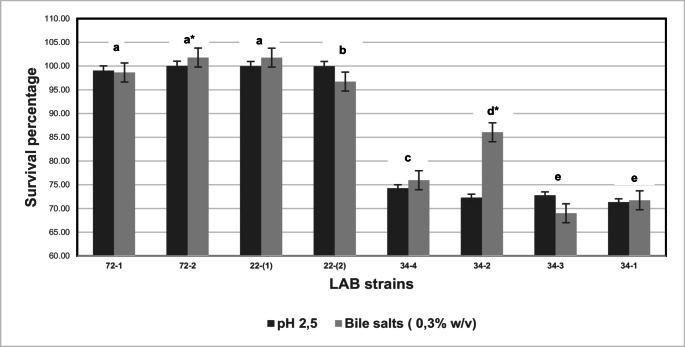
Survival percentages under simulated gastrointestinal conditions. Different letters indicate significant differences (*p* < 0.05) in survival percentages for each strain, and (*) indicates the difference among the strains according to their tolerance to simulated gastrointestinal conditions as per one-way ANOVA. The commercial bacteria were coded as follows according to their technical sheets: 34 − 4 (*L. acidophilus*), 34 − 2 (VEGE 092), 34 − 3 (*B. lactis*), 34 − 1 (*L. rhamnosus*)

Figure 1 shows the survival percentages under acidic conditions (pH = 2.5) and in the presence of bile salts (0.3%, w/v). No significant differences (*p* ≥ 0.05) were found between the two probiotic potential tests under simulated gastrointestinal tract conditions for the native strains; however, the strains did show significant differences among themselves. The strains that exhibited survival rates greater than 70% under acidic conditions were the commercial strain *L. plantarum* (74.27%) and the native strain *P. pentosaceus* isolate 22 − 1 (100.02%).

The best results regarding bile salt tolerance (0.3%) were observed for the commercial strain VEGE 092 (86.07%) and regarding the wild strain of *P. pentosaceus*, it exhibits a high acid tolerance and can survive in lower pH conditions (101.77%) (Fig. 1). Interestingly, the survival rate of* P. pentosaceus *under acidic conditions exceeded 100%, which indicates growth rather than mere survival. This resultd suggests that the strain may possess acid resilience and/or undergo acid-induced metabolic adaptations, aspects that merit further investigation. Our study found high tolerance to stress conditions of commercial strains used in plant substrates and recently isolated bacteria; however, strains isolated from bee bread presented values higher than 90% compared to non-native strains. Native strains are adapted to the environment in which they are found, which could improve their survival and effectiveness in specific conditions, such as the human gastrointestinal tract, by presenting greater genetic diversity and being less exposed to unwanted genetic modification that can be associated with strains of commercial origin [25], allowing the possibility of developing biotechnological applications, such as the production of functional foods.

The importance of substrate formulation for the inclusion of these cultures to maintain viability under sub-lethal stress conditions has been suggested. In vitro studies with *P. pentosaceus* strains subjected to lactic and acetic acid treatments demonstrated that stress under these conditions leads to growth stagnation and eventual cell death. However, supplementing the treatments with functional oligosaccharides, particularly xylooligosaccharides (XOS), maintained culture viability [26].

In this study, the two cultures with the highest percentages belong to the genus *Pediococcus* the literature mentions that bile salt tolerance varies by strain, ranging from approximately 0.1 to 1.0% [27]. However, the native strain presented a 100% survival rate, with a growth increase of 1 Log CFU/mL at 3 h of evaluation, which could indicate the potential BSH enzymatic activity.

### *In Vitro* Evaluation of Technological Potential

**Fig. 2:**
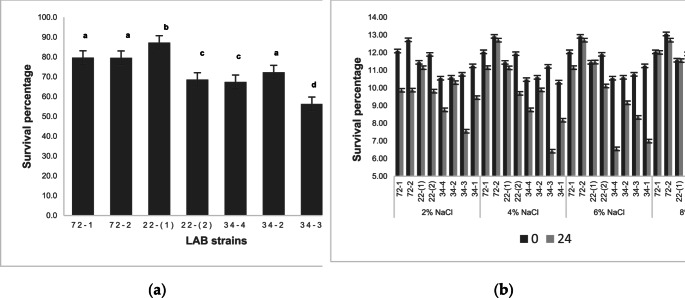
Survival percentages under simulated technological conditions. (**a**) High temperature tolerance (80 °C for 10 min); (**b**) Tolerance to high osmotic pressures. The statistical analysis to find significant differences (*p* < 0.05) was carried out according to the generalized linear model. The commercial bacteria were coded as follows according to their technical sheets: 34 − 4 (*L. acidophilus*), 34 − 2 (VEGE 092), 34 − 3 (*B. lactis*), 34 − 1 *(L. rhamnosus*)

In Fig. 2, the tolerance of high temperatures and high osmotic pressure is presented respectively.

Most of the strains evaluated had survival values higher than 70%, with the exception of the commercial strain *B. lactis*. The commercial strain with the highest percentage of tolerance to thermal shock was VEGE 092 (72.3%), for the native strain it was isolate 22 − 1 (*P. pentosaceus*) with a value of 87.3%. In the high salt concentration tolerance test, significant differences (*p* < 0.05) were found between the strains and the treatments evaluated with respect to the incubation time from 0 to 24 h. A similar behavior was observed for commercial cultures as for native strains. As the NaCl concentration increased, the viability at 24 h decreased by approximately 2 to 3 Log CFU/mL, with the particularity of isolate 22 − 1 (*P. pentosaceus*) having a viability of 11.73 ± 0.22 Log CFU/mL at 24 h of incubation at a salt concentration of 8% in MRS broth.

Fermentation temperature is one of the important factors that affect the viability of probiotic microorganisms, the favorable temperature for growth of most probiotics is in the range of 37–43 °C. Although certain species such as *L. acidophilus* can grow at temperatures up to 45 °C [5]. Not only the capability of the species but also of the strain and its relationship with the substrate to survive extreme temperature conditions must be considered.

*Pediococcus* species have shown growth in high salt concentrations and at different pH levels, implying a possible role in the fermentation of different salted meats, fermented vegetable products and in cheese ripening when pH decreases due to the growth of different starter cultures in elevated salt concentrations and reduced water activity [28]. This corresponds to various mechanisms of action such as the intracellular regulation of the concentration of certain amino acids (proline, glutamate and alanine) [29], however, the main response of lactic acid bacteria to osmotic stress is the intracellular accumulation of osmoprotectants, the most common being glycine betaine, choline, carnitine and dimethylsulfoniumacetate [30].

Considering the results described above on the probiotic and technological potential (in vitro), the work strains were selected. In the case of commercial cultures, the strain commercially identified as VEGE 092 (code 34 − 2) was chosen, and for the native strains, the isolate with code 22-(1), molecularly identified as *P. pentosaceus.*

### Growth of Lactic Acid Bacteria Strains on Alternative Substrates

**Fig. 3:**
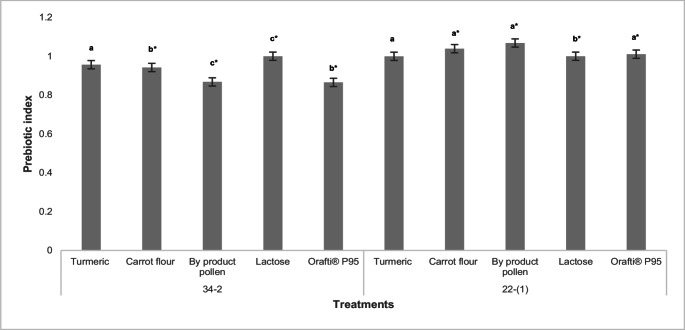
Prebiotic index of alternative substrates. Different letters represent significant differences (*p* < 0.05) between the substrates and the control (lactose) for each strain, and * indicates the difference between strains according to one-way ANOVA. Code 34 − 2 is the commercial strain (VEGE 092) and code 22-(1) is the native strain of *P. pentosaceus*

Figure 3 shows the prebiotic index of the different carbon sources evaluated for the selected strains.

The results of the prebiotic index indicated differences in growth between the strains and the different substrates evaluated (*p* ≤ 0.05). In the case of VEGE 092 (34 − 2), none of the alternative carbon sources, including the commercial prebiotic (Orafti P95), had an index equal to or higher than the value of lactose. However, for the native strain of *P. pentosaceus* it presented an opposite behavior, being the pollen cake (by-product of the extraction with supercritical fluid), the carbon source with the highest index with a value of 1.07.

These results reaffirm the metabolic superiority of native probiotic cultures over commercial bacteria, due to a better specific adaptation to the substrate by presenting specific capacities that allow them to resist the biochemical characteristics of a particular matrix, such as low pH, high content of phenolic compounds and other intrinsic properties. The origin of the isolate of the *P. pentosaceus* strain may be an essential factor in the growth in the pollen extraction by-product. “While comprehensive direct evidence on the broad prebiotic effect of whole bee pollen is still emerging, specific components or by-products, such as the pollen cake evaluated in this study, have shown promising prebiotic potential [31]. Recent studies have shown that phenolic compounds (flavonoids and phenolic acids) from ethanoic extracts of pollen (*Apis mellifera* carpatica) could be a rich source of prebiotic compounds, given their ability to stimulate the growth of microbial strains with probiotic potential (*L. rhamnosus* MF9 and *Enterobacter faecalis* 2M17), specifically, gallic acid and catechin could be considered growth-promoting agents for lactic acid bacteria [32].

Although the mechanisms underlying bee bread production are not fully elucidated, it has been assumed that lactic acid bacteria affect the pollen maturation process by reducing pH and modifying sugar and amino acid profiles [11]. *p*-Coumaric acid, a phenolic compound, is a structural component of sporopollenin, which constitutes the outer wall of the pollen grain. LAB can metabolize phenolic acids through strain-specific decarboxylase and/or reductase activity. In particular, caffeic and p-coumaric acids can be reduced respectively to dihydrocaffeic and phloretic acids, promoting cell growth [14].

The turmeric evaluated presented an index equal to that of lactose for *P. pentosaceus* and for VEGE 092, proving to be a carbon source for indigenous and commercial crops. The polyphenol curcumin accounts for 2–8% of most turmeric preparations and is generally considered to be its most active component, with antioxidant, anti-inflammatory, anticancer and potential prebiotic properties [33], driven by polysaccharide catabolism and sugar metabolism. Furthermore, the *Pediococcus* genus has the ability to ferment sugars directly associated with the metabolization of specific substrates [28]. Curcumin cannot function as a direct energy source for commensal microbiota and its effects are indirect based on alterations in host physiology, which may include changes in barrier function or through selective survival of bacteria [33].

The in vitro prebiotic potential of agro-industrial waste such as carrot flour was demonstrated (**Fig. 3**), according to the literature these wastes are considered low-cost prebiotic substrates, being an attractive strategy for added value and consequently mitigating environmental pollution associated with agro-industrial waste [34]. Studies have suggested that carrot dietary fiber (CDF) was more conducive to the growth of *L. rhamnosus* compared with phenol-free carrot dietary fiber (CDF-DF) [35], indicating that fiber-bound polyphenols might significantly contribute to the fermentation and antioxidant properties of dietary fiber in vivo and in vitrothrough the following mechanisms: (1) diversity in gut microbiota; (2) SCFA production and effects on pH; (3) antioxidant properties [35]. It should be noted that these findings are preliminary and based solely on in vitro assays; therefore, the absence of in vivo validation and limited safety data (e.g., antibiotic resistance or hemolytic activity) represent important limitations of this study. Future work should include comprehensive safety assessments and in vivo studies to confirm the functional potential of the strains and explore their applicability in product development. To facilitate the comparison of probiotic and technological properties among the strains, all results are summarized in Table 2.

**Table 2:** Summary of probiotic and technological properties of native and commercial lactic acid bacteria strains

StrainCode	Identity	GI Tolerance (%)	Technological Potential (%)	Prebiotic Index (Turmeric)	Prebiotic Index (Carrotflour)	PrebioticIndex (Beepollen)
22-(1)	*P. pentosaceus *(native)	65±0.10	60.0±0.04	1,00±0.02	1,04±0.01	1,07±0.01
22-(2)	Native isolate	60±0.07	55.0±0.012	-	-	-
72-1	Native isolate	55±0.15	50.0±0.04	-	-	-
72-2	Native isolate	58±0.01	52.0±0.05	-	-	-
34-1	*L. rhamnosus *(commercial)	75±0.08	69.1±0.05	-	-	-
34-2	VEGE 092(commercial)	80±0.11	73.1±0.05	0.96±0.02	0.94±0.01	0.87±0.02
34-3	B. lactis(commercial)	70±0.04	65.0±0.05	-	-	-
34-4	L. acidophilus (commercial)	78±0.05	71.0±0.05	-	-	-

Values represent mean survival percentages or prebiotic index (±SD) from threeindependent experiments, each performed in triplicate. GI Tolerance corresponds to theaverage of survival at pH 2.5 and in 0.3% (w/v) bile salts. Osmotic Mean is the arithmeticmean of survival measured at 2, 4, 6, and 8% NaCl. Technological Potential is defined here as the mean of Heat Tolerance and Osmotic Mean, providing a compositeindicator of technological robustness

## Conclusions

The study highlights the existence of significant variability in phenotypic characteristics between commercial and native probiotic strains isolated from bee bread. Molecular identification revealed differences between strains and the description provided by the technical data sheets, which underlines the importance of a detailed characterization to understand probiotic properties. In addition, we report the isolation of strains of the *Pediococcus* genus in bee bread samples, a group of bacteria traditionally not commonly associated with bee products. The selected strains VEGE 092 (commercial culture) and *P. pentosaceus* (native culture), showed a remarkable capacity to resist simulated gastrointestinal conditions and extreme environments with survival values higher than 80 and 95%, respectively. This resistance is crucial for its survival and functionality in the human digestive system and in various agro-industrial processes such as inclusion in alternative substrates of plant and beekeeping origin, the combination of VEGE 92 with turmeric and *P. pentosaceus* with pollen extraction by-products being the most effective, as demonstrated by the high prebiotic index (value equal to or greater than 1) compared to control carbohydrates such as lactose, these results being fundamental for the development of symbiotic functional foods and beverages. Future perspectives include conducting more intervention studies, either in vivo or focused on the development of a fermented product. Additionally, there is technological potential to supply these microorganisms to beekeepers as a supplement enriched with lactic acid bacteria (LAB) for *Apis* species.

## Data Availability

No datasets were generated or analysed during the current study.
